# Beyond immunoglobulin G: Dissecting the role of colostrum in programming early immune function in calves*

**DOI:** 10.3168/jdsc.2024-0733

**Published:** 2025-03-03

**Authors:** L. Rostoll-Cangiano, M. Cid de la Paz, J.F. Pierre

**Affiliations:** 1 Department of Animal and Dairy Sciences, University of Wisconsin–Madison, Madison, WI 53706; 2 Department of Nutritional Sciences, University of Wisconsin–Madison, Madison, WI 53706

## Abstract

Adequate colostrum intake is essential for neonatal calf health and development, not only reducing disease incidence but also promoting accelerated growth, earlier reproductive maturity, improved first-lactation milk yields, and lower culling rates. Although these advantages might be partly attributed to antibody-mediated disease prevention, emerging evidence highlights the critical role of colostrum's bioactive components in programming postnatal development. Colostrum's nutrient and bioactive composition is uniquely tailored to meet the developmental needs of newborn calves. Compared with mature milk, colostrum contains higher concentrations of proteins, fats, vitamins, and minerals, along with an array of growth factors and bioactive molecules that exert a foundational influence on the adaptation to extrauterine life. A deeper understanding of how these colostrum bioactive components collectively influence calf health is important to improve colostrum management and pave the way for refined nutritional and care strategies to support calves during this critical developmental period. In this review, we explore the multifaceted role of bovine colostrum beyond transfer of passive immunity, focusing on its influence in modulating early microbial encounters and guiding neonatal immune development.

## Highlights

• Colostrum effects extend beyond IgG, actively shaping immune development.
• Colostrum growth factors promote immune tolerance and intestinal maturation.
• Refined colostrum feeding strategies hold potential to enhance long-term health.

## Graphical Abstract

**Figure:**
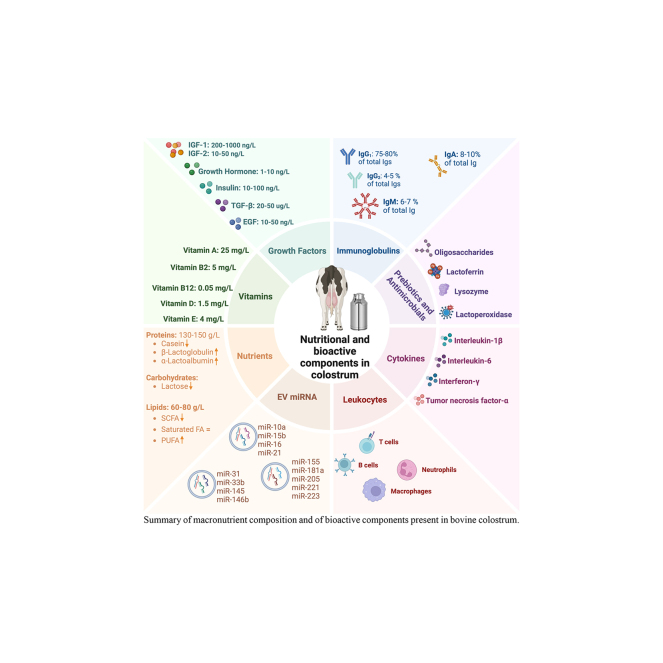
**Summary:** Colostrum's unique macronutrient and bioactive composition is specifically tailored to meet the neonatal calf's developmental needs. Colostrum contains a wide array of growth factors and bioactive molecules such as immunoglobulins (Ig), growth hormones, microRNA (miRNA), cytokines, antimicrobial peptides, oligosaccharides, and maternal immune cells, which exert a foundational influence on intestinal and immune development. A deeper understanding of how these colostrum bioactive components collectively influence calf health is important to improve colostrum management and pave the way for refined nutritional and care strategies to support calves during this critical developmental period.

Neonatal calves are particularly vulnerable to environmental pathogens in their early weeks of life, a period marked by elevated morbidity (35%–60%) and mortality (5%–8%) rates (Urie et al., 2018) in US dairy farms. The unique structure of the bovine placenta maintains maternal and fetal blood entirely separate, precluding in utero transfer of Ig and other immune factors. As a result, calves are born immunologically naive and depend on colostrum for immune protection during the critical first weeks of life (Windeyer and Gamsjäger, 2019). The transfer of Ig, known as transfer of passive immunity, is a key determinant of neonatal calf health and survival (Godden et al., 2019). Adequate colostrum intake has effects that extend well beyond the immediate reduction in disease incidence, and long-term benefits on health and productivity have been extensively reported in the literature (Faber et al., 2005; Abuelo et al., 2021). Calves with adequate intake of colostrum show accelerated growth, reach reproductive maturity earlier, produce more milk during their first lactation, and have lower culling rates (Faber et al., 2005; Abuelo et al., 2021). Although some of these benefits may result from the antibody-mediated reduction in disease, there is growing evidence that colostrum's array of growth factors and bioactive components contribute significantly to these outcomes. A deeper understanding of the broader role of colostrum on calf health and development holds valuable implications for farm management, offering opportunities to refine nutritional and care strategies that support calves through this vulnerable period. In this review, we discuss recent ruminant and nonruminant literature focused on the multifaceted role of colostrum beyond passive immunity transfer.

Newborn calves have underdeveloped gastrointestinal tract at birth, and colostrum's composition is adapted to deliver highly absorbable nutrients to support this developmental stage (Hammon and Blum, 1998; Liermann et al., 2020). Colostrum has a protein content of approximately 14% to 16%, more than 4-fold the concentration found in mature milk. In addition, colostrum has almost double the concentration of lipids compared with milk, with 6% to 7% depending on diet, breed, and time of the year (Westhoff et al., 2023). Furthermore, colostrum contains a significantly lower concentration of lactose, at around 2.5%, compared with approximately 5% in mature milk. This reduction in lactose helps reduce the osmotic pressure within the gut lumen, aiding in more efficient nutrient and Ig absorption (Wilms et al., 2019). Given the immature digestive system of neonatal calves, lower lactose concentrations help prevent diarrhea and support a smoother transition to enteral nutrition.

Colostrum also contains significantly higher concentrations of both fat-soluble vitamins, such as vitamins A, D, E, and K, and water-soluble vitamins, including vitamins B_1_, B_2_, B_6_, B_12_, and C (Kehoe et al., 2007). Vitamins A, E, and C counteract reactive oxygen species produced during the transition to the extrauterine environment, mitigating oxidative stress caused by the high-oxygen atmosphere and increased metabolic demands of thermoregulation and growth (Saugstad, 2003). Additionally, colostrum is a rich source of both enzymatic and nonenzymatic antioxidants, including superoxide dismutase, catalase, and glutathione peroxidase (Przybylska et al., 2007), which help limit oxidative damage. Studies show that colostrum feeding reduces oxidative stress markers in calves and increases antioxidant capacity, highlighting its role in protecting neonates against oxidative stress (Abuelo et al., 2014; Mann et al., 2023).

A defining feature of colostrum is its high concentration of Ig, particularly IgG, comprising up to 70% of the total protein in colostrum (Larson et al., 1980). Colostrum Ig confer protection against extracellular pathogens during the first 2 to 3 mo of life (Windeyer and Gamsjäger, 2019). During the first 24 h of life, the neonatal gut can absorb these Ig directly into the bloodstream, providing antibodies that protect against systemic infections (Godden et al., 2019). Although Ig absorption across the gut epithelium ceases after this initial window (Stott et al., 1979), colostrum-Ig continue to exert local effects by binding enteric pathogens and reducing their attachment to mucosal surfaces (Figure 1). In humans and mice, milk-derived Ig shape the intestinal microbiota by neutralizing harmful pathogens and promoting the colonization of beneficial microbes near the mucosal surfaces of the intestine (Donaldson et al., 2018). Although direct evidence in calves is limited, studies have shown that extended feeding of colostrum and transition milk can reduce the incidence and severity of diarrhea in preweaning calves (Berge et al., 2009; Kargar et al., 2020).

**Figure 1:**
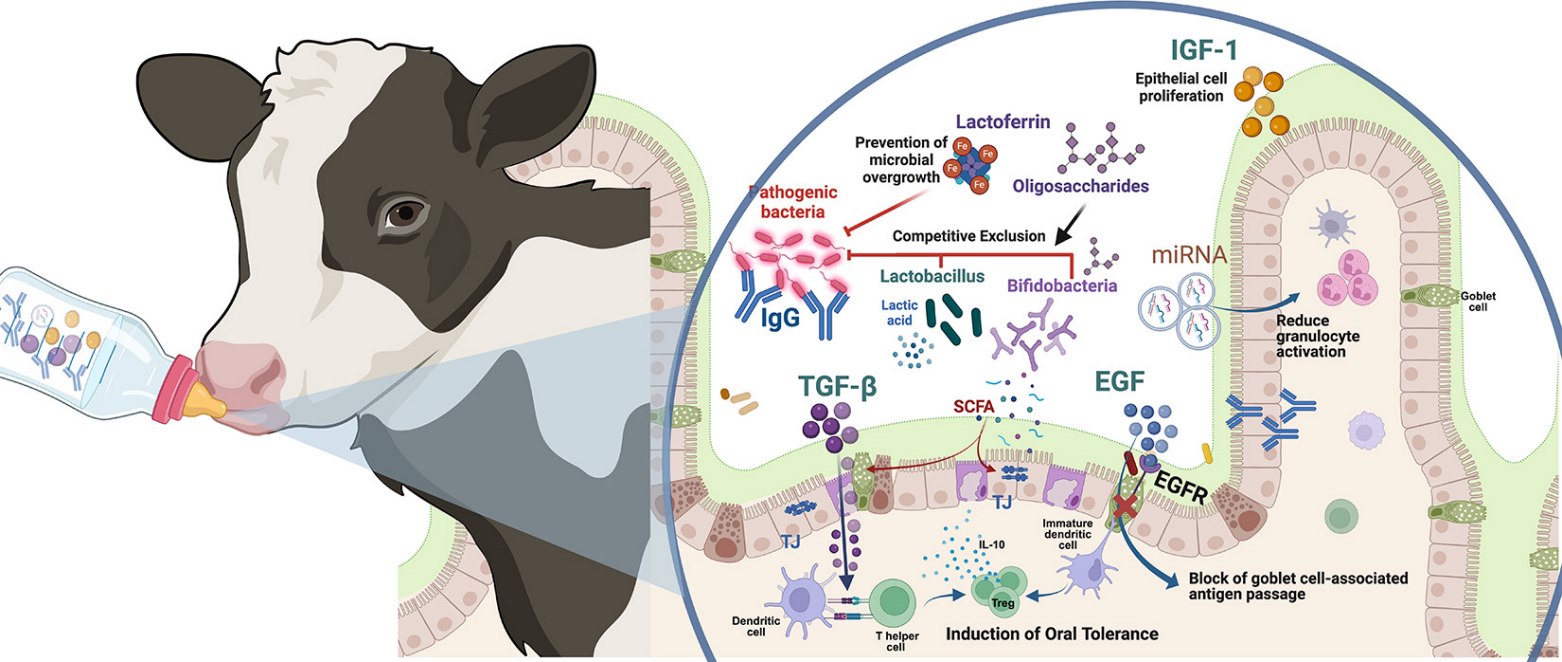
Colostrum and transition milk deliver bioactive components critical for programming intestinal immune development. Although Ig absorption across the gut epithelium is limited to the first 24 h of life, colostrum-derived immunoglobulins continue to exert local effects by binding enteric pathogens and preventing their attachment to mucosal surfaces. MicroRNAs help regulate inflammation by reducing granulocyte activation. Growth factors such as insulin-like growth factor 1 (IGF-1), transforming growth factor β (TGF-β), and insulin promote epithelial proliferation and mucus production, and mice models have shown that TGF-β and epidermal growth factor (EGF) support oral tolerance by driving naive T cell differentiation into regulatory T cells and blocking antigen translocation through goblet cell-associated antigen passages (GAPs). Oligosaccharides serve as prebiotics, fostering the growth of beneficial bacteria like *Bifidobacterium* and *Lactobacillus*, which ferment these compounds into SCFA and lactic acid. The SCFA stimulate anti-inflammatory cytokine production, such as IL-10, reinforcing oral tolerance, while lactic acid lowers the local pH, limiting bacterial growth near intestinal epithelial cells. TJ = tight junction. Figure created with BioRender.com.

At birth, calves experience a dramatic physiological transition from a sheltered in utero environment to an external world filled with microorganisms and environmental challenges—arguably one of the most profound shifts in the lifespan of any placental mammal. This transition places unique demands on the immune system, which must swiftly respond to protect the neonate from harmful pathogens while allowing tolerance of commensal microbes (Hoek et al., 2009). Therefore, establishing a balanced immune-microbial mutualism is critical to prevent unnecessary immune responses (Chase and Kaushik, 2019). The mucosal immune system in neonatal calves undergoes rapid development during the early postnatal period, as evidenced by transcriptome analyses of the small intestine (Malmuthuge et al., 2012; Liang et al., 2016). Within the first week postpartum, the expression of genes associated with tight junction proteins, antimicrobial peptides, B cell development, and regulatory T cell function exhibit a significant increase. Colostrum feeding plays a crucial role in modulating these pathways; delaying colostrum intake has been shown to impair intestinal development, reduce dendritic cell maturation, and increase granulocyte activation (Pyo et al., 2020; Song et al., 2021).

Beyond IgG, colostrum contains a wide range of high- and low-abundance proteins and complex carbohydrates that modulate intestinal development and early immune responses, facilitating the calf's adaptation to its new environment (Verhasselt, 2010; Torres-Castro et al., 2018). Bovine colostrum is enriched with a diverse array of growth factors including insulin-like growth factor-1 (**IGF-1**), growth hormone (**GH**), insulin, transforming growth factor-β (**TGF-β**), and epidermal growth factor (**EGF**). These trophic peptides promote rapid cell growth, differentiation, and maturation of the intestinal tract (Blum and Baumrucker, 2002). Although the roles of many of these factors in ruminants remain incompletely understood, mounting evidence suggests they contribute to the development of the gastrointestinal, endocrine, and immune systems (Chase et al., 2008; Hammon et al., 2020; Hare et al., 2023). The immature gastrointestinal tract of the neonate allows absorption of many of these bioactive molecules, often present in colostrum at concentrations exceeding those in maternal plasma, emphasizing their importance to neonatal development.

The trophic effects of GH, IGF-1, and insulin have been shown to significantly influence intestinal maturation in calves (Figure 1). Growth hormone exerts its effects through the stimulation of IGF-1 production, which facilitates epithelial cell growth and repair of the intestinal lining (Hammon et al., 2012, 2013). Insulin-like growth factor-1 also promotes the secretion of mucus and antimicrobial peptides, providing additional defense against luminal microbes (Hammon et al., 2012, 2013). Recently, colostrum insulin has been shown to promote small intestinal development because addition of insulin in colostrum increases villus height and mucus production (Hare et al., 2023). The intestinal barrier provides a physical separation between the intestinal immune system and the luminal environment, densely populated with microorganisms (Figure 1). By driving epithelial cell proliferation, enhancing mucus production, and stimulating the secretion of antimicrobial peptides, these growth factors contribute to the establishment of a protective layer that limits direct contact between microbes and epithelial cells. This separation is essential to avoid unnecessary immune responses to commensal microorganisms, thereby reducing potential pathological outcomes (Chase and Kaushik, 2019).

Milk TGF-β is found at concentrations 10- to 100-fold higher than those in human milk, being one of the main growth factors present in bovine colostrum (Oddy and Rosales, 2010). Much of the current understanding of its immunoregulatory properties, however, comes from mice and human studies. Transforming growth factor-β promotes epithelial cell proliferation and differentiation and also promotes oral tolerance in neonatal mice (Figure 1; Verhasselt, 2010; Torres-Castro et al., 2018) by driving the differentiation of naive T cells into regulatory T cells (**Treg**). Regulatory T cells are important for suppressing unnecessary immune responses against commensal microbes and dietary antigens (Huibregtse et al., 2007). In ruminants, however, studies suggest that Treg, although present, do not promote immune tolerance, at least in vitro (Hoek et al., 2009). Instead, these regulatory functions have been shown to be partially mediated by a subset of γδ T cells, which represent the predominant lymphocyte population in neonatal calves (Guzman et al., 2014; Cangiano et al., 2024). Notably, colostrum-deprived calves exhibit a significantly lower percentage of circulating γδ T cells within the lymphocyte population (Krueger et al., 2016), suggesting that colostrum may play a role in promoting the expansion of γδ T cells during the neonatal period. Future studies are needed to determine whether colostrum influences γδ T cell development to promote early immune tolerance and adaptation to the extrauterine environment in early postnatal life.

Another colostrum bioactive peptide that plays an important role in mediating oral tolerance is EGF (Knoop et al., 2020). This growth factor promotes cellular growth, proliferation, and differentiation of the intestinal epithelium (Figure 1; Torres-Castro et al., 2018), although is it found at lower concentrations than other growth factors in bovine colostrum (Blum and Baumrucker, 2002). In mice, EGF has been shown to protect against the development of necrotizing enterocolitis through inhibition of Toll-like receptor-4 and inhibit the translocation of *Escherichia coli* by preventing the formation of goblet cell-associated antigen passages (Good et al., 2015; Knoop et al., 2020). This protective mechanism is mediated through the activation of EGF receptor, effectively limiting bacterial dissemination from the gut to systemic circulation and reducing the risk of sepsis in neonatal mice.

Bovine colostrum is also a rich source of microRNAs (**miRNAs**), which mediate maternal-to-calf communication by fine-tuning the expression of several genes involved in immune function and intestinal development (Santoro et al., 2023). These small, noncoding RNAs are encapsulated within extracellular vesicles (**EV**), protecting them from enzymatic digestion and enabling their delivery to target cells (Baier et al., 2014). Interestingly, miRNA profiles differ across the lactation curve, suggesting the timing of consumption of colostrum, transitional milk, and mature milk has intentional purposes in shaping cellular response in the calf (Chen et al., 2010). Several hundred unique miRNAs have been identified in colostrum, with the *Let-7*, *miR-16*, and *miR-21* families being the most abundant (Li et al., 2012). Studies in murine models and neonatal calves indicate that these miRNA families influence the differentiation and functional development of the intestinal epithelium (McKenna et al., 2010; Liang et al., 2014). Additionally, supplementation of bovine EV to mice has anti-inflammatory effects, modulate cytokine production, and reduces activation in various myeloid and lymphoid cells in vitro (Arntz et al., 2015). In calves, these miRNAs are hypothesized to facilitate host-microbiota interactions by modulating the composition of beneficial microbial populations because the expression levels of several miRNAs in the intestine were positively correlated with the abundance of *Bifidobacterium* and *Lactobacillus* (Liang et al., 2014). Although the biological effects of dietary miRNAs in neonatal calves are not yet fully understood, emerging evidence underscores their importance in early-life development to help successfully cope with the extrauterine environment (Liang et al., 2014; Van Hese et al., 2020). Future research is needed to elucidate their mechanisms of absorption, the concentration thresholds required for biological activity, and their long-term effects on calf health.

Colostrum contains viable maternal leukocytes, including macrophages, neutrophils, natural killer cells, and T and B lymphocytes, and it has been reported that these cells can cross the intestinal barrier. In a series of elegant papers Reber et al. (2006; Reber et al., 2008a, Reber et al., 2008b explored the ability of maternal leukocytes to enter neonatal circulation and induce changes in immune function. They showed that colostrum induces phenotypic changes in maternal leukocytes to facilitate their trafficking across the calf's gut and demonstrated that colostrum leukocytes are absorbed and enter neonatal circulation (Reber et al., 2006). These cells peak in circulation within 12 to 24 h postingestion and are no longer detectable by 48 h. This rapid appearance and subsequent disappearance suggest that maternal cells migrate to peripheral lymph nodes. Evidence from murine models supports this hypothesis; breast-milk T cells express high levels of gut-homing receptors and localize to the Peyer's patches of nursed offspring (Cabinian et al., 2016). However, no evidence in ruminants is available to understand the full scope of their role in neonatal immune function. To what extent maternal immune cells in colostrum are important to confer protection to calves is inconclusive because most studies have only detected marginal differences in cell mediated immunity only lasting for the first weeks of life (Reber et al., 2008a,b).

Last, colostrum and, to a lower extent, transition milk contains a group of complex carbohydrates resistant to enzymatic digestion known as oligosaccharides (**OGS**; Fischer et al., 2018). Oligosaccharides are synthesized by epithelial cells of the mammary gland and secreted into colostrum to act as prebiotics stimulating the development of key bacterial species, such as *Bifidobacterium*, *Lactobacillus*, and *Bacteroides*. These bacteria possess a set of enzymes that allows them to ferment OGS into short-chain fatty acids (**SCFA**) and lactic acid (Fischer et al., 2018), which in turn stimulate production of the anti-inflammatory cytokines and promote induction or oral tolerance (Martens et al., 2008). Delaying colostrum or restricting colostrum feeding reduces microbial diversity and the abundance of these beneficial bacteria (Malmuthuge et al., 2015). In addition, *Bifidobacterium* and *Bacteroides* spp. can “forage” on mucin glycans and reside in the outer portion of the mucous layer and produce lactate, decreasing pH in the vicinity of epithelial cells, competitively excluding bacterial groups that cannot thrive in the acidic environment (Rios-Covian et al., 2013). Last, OGS can act as decoys that mimic host cell surface receptors, preventing pathogen attachment to intestinal epithelial cells (Kuntz et al., 2009), reducing the risk of infection.

In summary, bovine colostrum plays a vital role beyond passive immunity transfer, providing protection against infections while serving as a key driver of immune education. Evidence from both ruminant and nonruminant studies suggests that bovine colostrum supports neonatal immune development by promoting tolerance to commensal microorganisms, shaping microbial colonization, and mitigating excessive inflammatory and oxidative stress. This perspective underscores opportunities to refine nutritional management strategies for dairy calves to improve not only improve preweaning health and survival but also to support long-term health and productive outcomes.

## References

[bib1] Abuelo A., Cullens F., Brester J.L. (2021). Effect of preweaning disease on the reproductive performance and first-lactation milk production of heifers in a large dairy herd. J. Dairy Sci..

[bib2] Abuelo A., Perez-Santos M., Hernandez J., Castillo C. (2014). Effect of colostrum redox balance on the oxidative status of calves during the first 3 months of life and the relationship with passive immune acquisition. Vet. J..

[bib3] Arntz O.J., Pieters B.C.H., Oliveira M.C., Broeren M.G.A., Bennink M.B., de Vries M., van Lent P.L.E.M., Koenders M.I., van den Berg W.B., van der Kraan P.M., van de Loo F.A.J. (2015). Oral administration of bovine milk derived extracellular vesicles attenuates arthritis in two mouse models. Mol. Nutr. Food Res..

[bib4] Baier S.R., Nguyen C., Xie F., Wood J.R., Zempleni J. (2014). MicroRNAs are absorbed in biologically meaningful amounts from nutritionally relevant doses of cow milk and affect gene expression in peripheral blood mononuclear cells, HEK-293 kidney cell cultures, and mouse livers. J. Nutr..

[bib5] Berge A.C.B., Besser T.E., Moore D.A., Sischo W.M. (2009). Evaluation of the effects of oral colostrum supplementation during the first fourteen days on the health and performance of preweaned calves. J. Dairy Sci..

[bib6] Blum J.W., Baumrucker C.R. (2002). Colostral and milk insulin-like growth factors and related substances: Mammary gland and neonatal (intestinal and systemic) targets. Domest. Anim. Endocrinol..

[bib7] Cabinian A., Sinsimer D., Tang M., Zumba O., Mehta H., Toma A., Sant'Angelo D., Laouar Y., Laouar A. (2016). Transfer of maternal immune cells by breastfeeding: Maternal cytotoxic T lymphocytes present in breast milk localize in the Peyer's patches of the nursed infant. PLoS One.

[bib8] Cangiano L.R., Lamers K., Olmeda M.F., Villot C., Hodgins D.C., Mallard B.A., Steele M.A. (2024). Developmental adaptations of γδ T cells and B cells in blood and intestinal mucosa from birth until weaning in Holstein bull calves. J. Dairy Sci..

[bib9] Chase C., Kaushik R.S. (2019). Mucosal immune system of cattle: All immune responses begin here. Vet. Clin. North Am. Food Anim. Pract..

[bib10] Chase C.C.L., Hurley D.J., Reber A.J. (2008). Neonatal immune development in the calf and its impact on vaccine response. Vet. Clin. North Am. Food Anim. Pract..

[bib11] Chen X., Gao C., Li H., Huang L., Sun Q., Dong Y., Tian C., Gao S., Dong H., Guan D., Hu X., Zhao S., Li L., Zhu L., Yan Q., Zhang J., Zen K., Zhang C.-Y. (2010). Identification and characterization of microRNAs in raw milk during different periods of lactation, commercial fluid, and powdered milk products. Cell Res..

[bib12] Donaldson G.P., Ladinsky M.S., Yu K.B., Sanders J.G., Yoo B.B., Chou W.-C., Conner M.E., Earl A.M., Knight R., Bjorkman P.J., Mazmanian S.K. (2018). Gut microbiota utilize immunoglobulin A for mucosal colonization. Science.

[bib13] Faber S.N., Faber N.E., Mccauley T.C., Ax R.L. (2005). Case study: Effects of colostrum ingestion on lactational performance. Prof. Anim. Sci..

[bib14] Fischer A.J., Malmuthuge N., Guan L.L., Steele M.A. (2018). *Short communication:* The effect of heat treatment of bovine colostrum on the concentration of oligosaccharides in colostrum and in the intestine of neonatal male Holstein calves. J. Dairy Sci..

[bib15] Godden S.M., Lombard J.E., Woolums A.R. (2019). Colostrum management for dairy calves. Vet. Clin. North Am. Food Anim. Pract..

[bib16] Good M., Sodhi C.P., Egan C.E., Afrazi A., Jia H., Yamaguchi Y., Lu P., Branca M.F., Ma C., Prindle T., Mielo S., Pompa A., Hodzic Z., Ozolek J.A., Hackam D.J. (2015). Breast milk protects against the development of necrotizing enterocolitis through inhibition of Toll-like receptor 4 in the intestinal epithelium via activation of the epidermal growth factor receptor. Mucosal Immunol..

[bib17] Guzman E., Hope J., Taylor G., Smith A.L., Cubillos-Zapata C., Charleston B. (2014). Bovine γδ T cells are a major regulatory T cell subset. J. Immunol..

[bib18] Hammon H.M., Blum J.W. (1998). Metabolic and endocrine traits of neonatal calves are influenced by feeding colostrum for different durations or only milk replacer. J. Nutr..

[bib19] Hammon H.M., Liermann W., Frieten D., Koch C. (2020). Review: Importance of colostrum supply and milk feeding intensity on gastrointestinal and systemic development in calves. Animal.

[bib20] Hammon H.M., Steinhoff-Wagner J., Flor J., Schönhusen U., Metges C.C. (2013). Lactation Biology Symposium: Role of colostrum and colostrum components on glucose metabolism in neonatal calves. J. Anim. Sci..

[bib21] Hammon H.M., Steinhoff-Wagner J., Schönhusen U., Metges C.C., Blum J.W. (2012). Energy metabolism in the newborn farm animal with emphasis on the calf: Endocrine changes and responses to milk-born and systemic hormones. Domest. Anim. Endocrinol..

[bib22] Hare K.S., Wood K.M., Mustapha Y., Swanson K.C., Steele M.A. (2023). Colostrum insulin supplementation to neonatal Holstein bulls affects small intestinal histomorphology, mRNA expression, and enzymatic activity with minor influences on peripheral metabolism. J. Dairy Sci..

[bib23] Hoek A., Rutten V.P.M.G., Kool J., Arkesteijn G.J.A., Bouwstra R.J., Van Rhijn I., Koets A.P. (2009). Subpopulations of bovine WC1^+^ γδ T cells rather than CD4^+^ CD25^high^ Foxp3^+^ T cells act as immune regulatory cells ex vivo. Vet. Res..

[bib24] Huibregtse I.L., Snoeck V., de Creus A., Braat H., de Jong E.C., van Deventer S.J.H., Rottiers P. (2007). Induction of ovalbumin-specific tolerance by oral administration of *Lactococcus lactis* secreting ovalbumin. Gastroenterology.

[bib25] Kargar S., Roshan M., Ghoreishi S.M., Akhlaghi A., Kanani M., Abedi Shams-Abadi A.R., Ghaffari M.H. (2020). Extended colostrum feeding for 2 weeks improves growth performance and reduces the susceptibility to diarrhea and pneumonia in neonatal Holstein dairy calves. J. Dairy Sci..

[bib26] Kehoe S.I., Jayarao B.M., Heinrichs A.J. (2007). A survey of bovine colostrum composition and colostrum management practices on Pennsylvania dairy farms. J. Dairy Sci..

[bib27] Knoop K.A., Coughlin P.E., Floyd A.N., Ndao I.M., Hall-Moore C., Shaikh N., Gasparrini A.J., Rusconi B., Escobedo M., Good M., Warner B.B., Tarr P.I., Newberry R.D. (2020). Maternal activation of the EGFR prevents translocation of gut-residing pathogenic *Escherichia coli* in a model of late-onset neonatal sepsis. Proc. Natl. Acad. Sci. USA.

[bib28] Krueger L.A., Beitz D.C., Humphrey S.B., Stabel J.R. (2016). Gamma delta T cells are early responders to *Mycobacterium avium* ssp. *paratuberculosis* in colostrum-replete Holstein calves. J. Dairy Sci..

[bib29] Kuntz S., Kunz C., Rudloff S. (2009). Oligosaccharides from human milk induce growth arrest via G2/M by influencing growth-related cell cycle genes in intestinal epithelial cells. Br. J. Nutr..

[bib30] Larson B.L., Heary H.L., Devery J.E. (1980). Immunoglobulin production and transport by the mammary gland. J. Dairy Sci..

[bib31] Li Z., Lan X., Guo W., Sun J., Huang Y., Wang J., Huang T., Lei C., Fang X., Chen H. (2012). Comparative transcriptome profiling of dairy goat microRNAs from dry period and peak lactation mammary gland tissues. PLoS One.

[bib32] Liang G., Malmuthuge N., Bao H., Stothard P., Griebel P.J., Guan L.L. (2016). Transcriptome analysis reveals regional and temporal differences in mucosal immune system development in the small intestine of neonatal calves. BMC Genomics.

[bib33] Liang G., Malmuthuge N., McFadden T.B., Bao H., Griebel P.J., Stothard P., Guan L.L. (2014). Potential regulatory role of microRNAs in the development of bovine gastrointestinal tract during early life. PLoS One.

[bib34] Liermann W., Schäff C.T., Gruse J., Derno M., Weitzel J.M., Kanitz E., Otten W., Hoeflich A., Stefaniak T., Sauerwein H., Bruckmaier R.M., Gross J.J., Hammon H.M. (2020). Effects of colostrum instead of formula feeding for the first 2 days postnatum on whole-body energy metabolism and its endocrine control in neonatal calves. J. Dairy Sci..

[bib35] Malmuthuge N., Chen Y., Liang G., Goonewardene L.A., Guan L.L. (2015). Heat-treated colostrum feeding promotes beneficial bacteria colonization in the small intestine of neonatal calves. J. Dairy Sci..

[bib36] Malmuthuge N., Li M., Fries P., Griebel P.J., Guan L.L. (2012). Regional and age dependent changes in gene expression of Toll-like receptors and key antimicrobial defence molecules throughout the gastrointestinal tract of dairy calves. Vet. Immunol. Immunopathol..

[bib37] Mann S., Gandy J., Curone G., Abuelo A. (2023). The effect of heat treatment on colostral and newborn calf redox status and oxylipid biomarkers. J. Dairy Sci..

[bib38] Martens E.C., Chiang H.C., Gordon J.I. (2008). Mucosal glycan foraging enhances fitness and transmission of a saccharolytic human gut bacterial symbiont. Cell Host Microbe.

[bib39] McKenna L.B., Schug J., Vourekas A., McKenna J.B., Bramswig N.C., Friedman J.R., Kaestner K.H. (2010). MicroRNAs control intestinal epithelial differentiation, architecture, and barrier function. Gastroenterology.

[bib40] Oddy W.H., Rosales F. (2010). A systematic review of the importance of milk TGF-β on immunological outcomes in the infant and young child. Pediatr. Allergy Immunol..

[bib41] Przybylska J., Albera E., Kankofer M. (2007). Antioxidants in bovine colostrum. Reprod. Domest. Anim..

[bib42] Pyo J., Hare K., Pletts S., Inabu Y., Haines D., Sugino T., Guan L.L., Steele M. (2020). Feeding colostrum or a 1:1 colostrum:milk mixture for 3 days postnatal increases small intestinal development and minimally influences plasma glucagon-like peptide-2 and serum insulin-like growth factor-1 concentrations in Holstein bull calves. J. Dairy Sci..

[bib43] Reber A.J., Donovan D.C., Gabbard J., Galland K., Aceves-Avila M., Holbert K.A., Marshall L., Hurley D.J. (2008). Transfer of maternal colostral leukocytes promotes development of the neonatal immune system: I. Effects on monocyte lineage cells. Vet. Immunol. Immunopathol..

[bib44] Reber A.J., Donovan D.C., Gabbard J., Galland K., Aceves-Avila M., Holbert K.A., Marshall L., Hurley D.J. (2008). Transfer of maternal colostral leukocytes promotes development of the neonatal immune system. Part II. Effects on neonatal lymphocytes. Vet. Immunol. Immunopathol..

[bib45] Reber A.J., Lockwood A., Hippen A.R., Hurley D.J. (2006). Colostrum induced phenotypic and trafficking changes in maternal mononuclear cells in a peripheral blood leukocyte model for study of leukocyte transfer to the neonatal calf. Vet. Immunol. Immunopathol..

[bib46] Rios-Covian D., Arboleya S., Hernandez-Barranco A.M., Alvarez-Buylla J.R., Ruas-Madiedo P., Gueimonde M., de los Reyes-Gavilan C.G. (2013). Interactions between *Bifidobacterium* and *Bacteroides* species in cofermentations are affected by carbon sources, including exopolysaccharides produced by bifidobacteria. Appl. Environ. Microbiol..

[bib47] Santoro J., Mukhopadhya A., Oliver C., Brodkorb A., Giblin L., O'Driscoll L. (2023). An investigation of extracellular vesicles in bovine colostrum, first milk and milk over the lactation curve. Food Chem..

[bib48] Saugstad O.D. (2003). Oxygen toxicity at birth: The pieces are put together. Pediatr. Res..

[bib49] Song Y., Sun H., He Z., Fischer-Tlustos A., Ma T., Steele M., Guan L.L. (2021). Transcriptome analysis revealed that delaying first colostrum feeding postponed ileum immune system development of neonatal calves. Genomics.

[bib50] Stott G.H., Marx D.B., Menefee B.E., Nightengale G.T. (1979). Colostral immunoglobulin transfer in calves I. Period of absorption. J. Dairy Sci..

[bib51] Torres-Castro P., Abril-Gil M., Rodríguez-Lagunas M.J., Castell M., Pérez-Cano F.J., Franch À. (2018). TGF-β2, EGF, and FGF21 growth factors present in breast milk promote mesenteric lymph node lymphocytes maturation in suckling rats. Nutrients.

[bib52] Urie N.J., Lombard J.E., Shivley C.B., Kopral C.A., Adams A.E., Earleywine T.J., Olson J.D., Garry F.B. (2018). Preweaned heifer management on US dairy operations: Part V. Factors associated with morbidity and mortality in preweaned dairy heifer calves. J. Dairy Sci..

[bib53] Van Hese I., Goossens K., Vandaele L., Opsomer G. (2020). *Invited review:* MicroRNAs in bovine colostrum—Focus on their origin and potential health benefits for the calf. J. Dairy Sci..

[bib54] Verhasselt V. (2010). Neonatal tolerance under breastfeeding influence: The presence of allergen and transforming growth factor-β in breast milk protects the progeny from allergic asthma. J. Pediatr..

[bib55] Westhoff T.A., Overton T.R., Mann S. (2023). Epidemiology of bovine colostrum production in New York Holstein herds: Prepartum nutrition and metabolic indicators. J. Dairy Sci..

[bib56] Wilms J., Berends H., Martín-Tereso J. (2019). Hypertonic milk replacers increase gastrointestinal permeability in healthy dairy calves. J. Dairy Sci..

[bib57] Windeyer M.C., Gamsjäger L. (2019). Vaccinating calves in the face of maternal antibodies. Vet. Clin. North Am. Food Anim. Pract..

